# Neither epithelial nor mesenchymal circulating tumor cells isolated from breast cancer patients are tumorigenic in NOD-*scid Il2rg*^*null*^ mice

**DOI:** 10.1038/npjbcancer.2016.4

**Published:** 2016-03-09

**Authors:** Vera S Donnenberg, Alexander Huber, Per Basse, J Peter Rubin, Albert D Donnenberg

**Affiliations:** 1 Department of Cardiothoracic Surgery, University of Pittsburgh School of Medicine, Pittsburgh, PA, USA; 2 University of Pittsburgh Cancer Institute, Pittsburgh, PA, USA; 3 McGowan Institute of Regenerative Medicine, Pittsburgh, PA, USA; 4 LifeNet Health, Virginia Beach, VA, USA; 5 Department of Immunology, University of Pittsburgh School of Medicine, Pittsburgh, PA, USA; 6 Department of Plastic Surgery, University of Pittsburgh School of Medicine, Pittsburgh, PA, USA; 7 Department of Medicine, Division of Hematology/Oncology, University of Pittsburgh School of Medicine, Pittsburgh, PA, USA

## Abstract

The quantitative evaluation of circulating EpCAM+ tumor cells (CTCs) in the peripheral blood of breast cancer patients provides an independent predictor of risk of progression in patients with metastatic disease. The present study investigated the tumorigenic potential of CTCs from cryopreserved mobilized leukapheresis products obtained from three metastatic breast cancer patients in remission. Cells were immunomagnetically separated if they expressed either the epithelial cell surface marker EpCAM, or CD90, a mesenchymal stromal cell marker associated with tumorigenic stem-like cancer cells. Cells were injected into the mammary fat pads of NOD-scid Il2rg^null^ mice. The injection of very large numbers of CTCs (0.3–1.5×10^6^ CTCs per site, 20 sites per sample) in an optimized xenograft model did not result in the establishment of human-derived tumor xenografts. Four orders of magnitude fewer cells of the same CD90+ phenotype, but obtained from metastatic breast cancer pleural effusions, were highly tumorigenic in the same model system. These results favor the interpretation that circulating tumor cell load does not directly bear on metastatic potential, and that tumorigenic circulating breast cancer cells in patients with metastatic breast cancer are exceedingly rare. Furthermore, the CD44+/CD90+ phenotypic signature indicative of tumorigenicity in cells separated from metastatic or primary breast tumors does not have the same significance in circulating tumor cells.

## Introduction

The metastatic spread of a primary tumor through the dissemination, seeding, and spreading of metastasis-inducing cells to a new anatomical site^1^ is the leading cause of cancer-related deaths in the United States.^2^ Whether metastasis-inducing cells first travel through the lymphatics or intravasate directly, hematogenous spread is required for distant metastasis. The quantitative evaluation of circulating EpCAM+ tumor cells (CTCs) in the peripheral blood of breast cancer patients provides an independent predictor of risk of progression in patients with metastatic disease.^3^ Despite the fact that circulating tumor cell burden has been proposed as a prognostic indicator, it is an independent predictor of progression and survival only in breast cancer patients who have already been diagnosed with metastatic disease.^3,4^ CTCs are commonly detectable in patients with early stage disease, but metastatic spread often takes years to manifest, making tumorigenic take of blood borne tumor cells a rare event. This may be a function of the properties of the CTCs themselves, the niche that they encounter, or a combination of both.

Baccelli *et al.* ^5^ demonstrated tumorigenic CTCs in a small proportion of breast cancer patients with very high CTC counts. In a series of 350 patients, peripheral blood from 110 patients was depleted of CD45+, CD66b+, and glycophorin A+ hematopoietic cells and tested for tumorigenicity in immunodeficient mice. Samples from three patients yielded tumors. The EpCAM+ CD44+ CD47+ MET+ phenotype that these authors attributed to tumorigenic circulating cells was not directly demonstrated. Rather, it was inferred from a correlation of the prevalence of this subset with disease progression in eight patients, and from the phenotype of the resulting xenografts, which were MET+. The EpCAM+ CD44+ CD47+ MET+ phenotype is common and was found in the circulation of all patients at varying frequency, including the vast majority whose circulating tumor cells were not tumorigenic. Furthermore, the patient samples that yielded tumors had extraordinarily high peripheral CTC counts (260–200,000 CTC/7.5 ml blood) as defined by Veridex CellSearch, and yielded 2,000–170,000 non-hematopoietic cells for tumorigenicity studies. For comparison, 5 CTC/7.5 ml blood is considered an indicator of poor prognosis in patients with metastatic breast cancer.^3^ As all samples were obtained from patients with preexisting metastatic disease, the results could equally well be interpreted to suggest that metastatic lesions in a small proportion of these patients (~2.7%) shed tumorigenic cells of uncharacterized phenotype into the blood. This must not be confused with the scenario in which a primary tumor sheds cells capable of metastatic spread.

The present study investigated the tumorigenic potential of circulating EpCAM+ and CD90+ cells isolated from mobilized leukapheresis products from three breast cancer patients collected for autologous hematopoietic stem cell transplantation. These graft products were collected as backups for CD34 enriched grafts and would have been used in the case of graft failure. Putative CTCs were isolated immunomagnetically based on the expression of either the epithelial cell surface marker EpCAM,^6,7^ or CD90. CD90 expression is associated with primitive hematopoietic progenitor cells, normal mesenchymal stromal cells and tumor cells that have undergone the epithelial-to-mesenchymal transition,^8–10^ a process well implicated in cancer metastasis.^11,12^ This inclusive cell collection strategy was optimized for cell recovery, including cells expressing low levels of EpCAM and/or CD90. Cells were injected into the mammary fat pads of NOD-scid Il2rg^null^ mice, an established mouse model for tumorigenicity of isolated human breast cancer cell subsets.^13,14^

## Results

**Figure 1:**
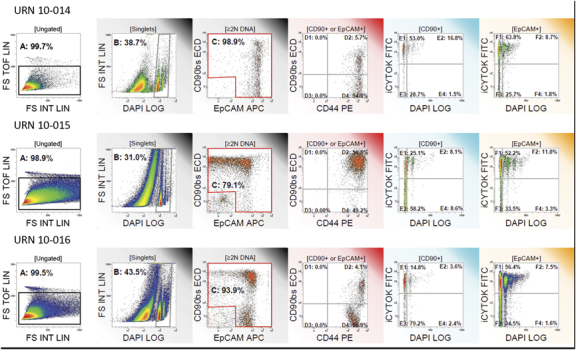
Flow cytometric analysis of leukapheresis cells selected for expression of EpCAM or CD90. From left to right: (**a**) Doublet discrimination limits analysis to singlet cells; (**b**) Subcellular debris and apoptotic cells are removed by restricting analysis to events having DNA content ⩾2N; (**c**) Assessment of Purity, the percent of cells expressing EpCAM and/or CD90 is indicated; (**d**) CD44 and CD90 among cells expressing EpCAM and/or CD90; (**e**) Ploidy and cytokeratin expression among all CD90+ cells. The demarcation between 2N and >2N DNA content was determined on the lymphocyte DNA peak in the preseparation samples (not shown); (**f**) Ploidy and cytokeratin expression among all EpCAM+ cells.

Patient peripheral blood counts rebounded after mobilization, and thawed leukapheresis products were viable, even after recovery from high-dose consolidation chemotherapy (Table 1). An average of 2×10^9^ viable cells was isolated from the three patients’ cryopreserved leukapheresis products after thawing and centrifuged using gradient centrifugation. Trypan blue viability was 60±14% (mean, s.d.). Subsequent immunomagnetic separation resulted in the recovery of a total of 6.0–36.1×10^6^ (range) putative CTCs per leukapheresis sample. The purity of separated cells was 90.6±10.3% (mean, s.d.; Figure 1c, Table 2). Post-separation flow cytometric analysis revealed that the most prevalent subset was EpCAM+ CD90− (74.0±30.4% of all isolated cells), but CD90+ cells (EpCAM+ and EpCAM−) comprised 4.4±1.5 and 12.4±19.9%, respectively. The CD44+/CD90+ phenotype, associated with tumorigenic breast cancer cells was present in 20.2±26.5% of selected cells (Figure 1d). This average was heavily influenced by the high CD44+/CD90+ content of patient URN 10-015’s selected cells (50.8%). This patient did not receive mobilization chemotherapy. Expression of cytokeratin among the selected cells, and the presence of cytokeratin+ aneuploid cells (>2N DNA content) among both CD90+ and EpCAM+ populations from all patients confirms the tumor origin of the cytokeratin+ and/or aneuploid subpopulations (Figures 1e and f). Virtually all of the EpCAM+ and/or CD90+ selected cells coexpressed CD44, a marker associated with epithelial-to-mesenchymal transition^11,15^ and tumorigenic breast cancer stem cells,^16^ and CD146 (not shown, 83.5±9.8%), a marker associated with epithelial-to-mesenchymal transition in breast cancer.^17^

Each immunomagnetically separated patient sample was divided into 20 equal inocula and injected into the mammary fat pads of NOD-*scid Il2rg*^*null*^ mice (four injections/mouse, five mice per sample). A total number of 0.3×10^6^, 1.3×10^6^, and 1.5×10^6^ CTCs were directly injected per site, respectively, maximizing the sensitivity to detect tumorigenicity of a rare subset among CTCs (Figure 1). Cells were admixed with first passage adipose stromal cells (ASC), (10,000 per injection) to maximize tumor cell engraftment as previously described^14^ and suspended in Matrigel to immobilize the xenograft at the site of injection and provide an optimal environment for tumor cell growth.^18^ Graded numbers (10–1,000,000 in log_10_ increments) of the hormone receptor positive breast cancer cell line BT-474, and ASC alone were suspended in Matrigel and injected into separate groups of mice as positive and negative controls, respectively.

**Figure 2:**
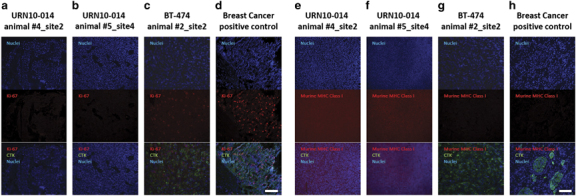
Immunofluorescent staining for human-specific Ki-67, human-specific cytokeratin, and murine major histocompatibility complex (MHC) Class I. Nuclei were stained with DAPI. Observed neoplasms in the URN10-014 group were negative for human Ki-67+ and human-specific cytokeratin (columns **a**,**b**), but stained positively for murine-specific MHC Class I (**e**,**f**). A BT474 xenograft (**c**,**g**) and a human metastatic breast cancer control (**d**,**h**) were positive for human cytokeratin and Ki-67, but negative for murine MHC Class I antigen. Scale bar (white)=100 μm.

BT-474 control injections resulted in the rapid formation of palpable tumors; mice were killed at 6 weeks, as warranted by tumor size of the mice receiving the highest dose. At necropsy, tumors were detected in a proportion of animals receiving as few as 10 BT-474 cells per site (Table 3). All remaining mice were killed at 6 months after injection. At the time of sacrifice, two animals from a single-treatment group (URN10-014) evidenced palpable tumors, both of which proved to be of murine origin (Figure 2), as determined by immunohistochemical staining with anti-murine major histocompatibility complex class I. None of the remaining animals in this group, none of the animals of the two other treatment groups (URN10-015, URN10-016), and none of the animals receiving ASC alone, evidenced tumors by macroscopic or histologic evaluation of the injection sites. Using the identical xenograft model, we have previously shown that mice injected with only 100 FACSorted CD90+ yielded tumors in almost half of the injection sites^14^ (Table 3).

## Discussion

Consistent with our previous observations on long-term cryopreserved hematopoietic stem cell products,^19^ cryopreserved leukapheresis products were highly viable, even in the two patients receiving mobilization chemotherapy. The present study demonstrates that viable CTCs were abundant in leukapheresis products collected from late-stage breast cancer patients in remission. It is not surprising that mobilization therapy failed to ablate circulating cytokeratin+ cells, particularly those expressing CD90+. We have previously observed that EpCAM+ CD44+ CD90+ breast carcinoma cells in a metastatic pleural effusion survived preferentially after palliative chemotherapy.^20^ It is therefore of great importance to determine whether this same phenotypic population, when isolated from the blood, is tumorigenic. Neither EpCAM+ CTCs nor circulating CD90+ cells isolated from these patients were tumorigenic in an optimized xenograft model. Optimization consisted of use of the most permissive immune deficient mice (NSG,^21^), orthotopic injection of CTC into the mammary fatpad, the creation of a conditioned microenvironment consisting of matrigel tumor extracellular matrix^18^ and coinjected ASC. ASC, and closely related mesenchymal stromal cells, have been shown to promote engraftment and tumor growth.^14,22–24^ In the present study, this environment was so conducive to tumor growth that spontaneous murine tumors, probably lymphomas, in two animals near the injection sites (Figure 2). Most importantly, the selected CTC were injected in 20 orthotopic replicate sites per patient at cell doses four orders of magnitude higher than those that we used to demonstrate tumorigenicity of sorted CD44+/CD90+ metastatic breast cancer cells in the identical animal model.^14^ This extraordinary cell dose was possible because the unique leukapheresis products allowed us to load an average of 2 billion cells/sample on the separation columns. Our post-separation characterization demonstrated that a significant proportion of the separated cells coexpressed cytokeratin, CD44, and CD146 and, by virtue of their epithelial-to-mesenchymal transition phenotype, might have been expected *a priori* to be good candidates for metastasis-inducing cells. Certainly tumor cells isolated from primary breast tumors^16^ or metastatic pleural effusions ^13,16,25^ bearing the same phenotypic profile would have been expected to induce tumors in this experimental system, especially when injected at such high cell dose.

Although we were able to study only three patient samples, the data strongly suggest that the great majority of CTCs are not tumorigenic in the best model for human tumorigenicity that is available at this time. These findings do not vitiate the utility of CTC quantification as a prognostic tool in the appropriate patient populations, but favor the interpretations that: (1) In most patients the majority of circulating tumor cells are unrelated to metastatic potential; and (2) although the existence of primary tumor-derived metastasis-inducing cells can be inferred from the natural history of breast cancer, it is presently unknown whether they have a unique phenotype. We suggest that a more likely interpretation of the Baccelli data are that, in a small proportion of patients with end-stage metastatic breast cancer and very high numbers of CTCs, the metastasis itself sheds tumorigenic tumor cells. This phenomenon is likely unrelated to the biologically and clinically significant process by which a primary tumor sheds cells capable seeding distant neoplasms.

## Materials and methods

### Patient samples

Participants provided written informed consent for the original study, which was a therapeutic trial including collection of ancillary specimens for research, under a protocol approved by the University of Pittsburgh Institutional Review Board (IRB, UPCI 93-73). Leukapheresis samples (*n*=3) were unused cryopreserved backup products for autologous stem cell grafts. They were held in the UPMC Hematopoietic Stem Cell Laboratory Cryopreservation Facility and were deaccessioned according to institutional criteria which include signoff by the laboratory director, medical director, and attending physician or clinical program director. They were released for research purposes under an IRB approved honest broker system. All patients had metastatic disease with histologically negative bone marrows at the time of sample collection and underwent leukapheresis after mobilization with G-CSF (5–15 μg/kg per day). Two patients received mobilization chemotherapy in combination with G-CSF (Table 1). Product collection was performed when the white blood corpuscles reached 1,000/mm^3^ for three consecutive days. Two patients (URN 10–14, URN 10–16) were deceased at the time of this study and had relapsed with metastatic breast cancer.

### Immunomagnetic enrichment of EpCAM+ and CD90+ cells

Cryopreserved cells were carefully thawed, heparinized (10 U/ml bovine lung heparin), transferred into 50-ml polypropylene conical tubes, and washed in ice-cold phosphate-buffered saline with 25% calf serum. Washed cells were incubated in buffer and DNase (350 KU/ml, Sigma-Aldrich, St Louis, MO, USA, Cat. No. D-5025), washed again and separated by Ficoll-Hypaque density-gradient separation (Histopaque 1077, Sigma-Aldrich). EpCAM+ and CD90+ cells were enriched by magnetic bead separation using the AutoMACS system (Miltenyi Biotech, Bergisch Gladbach, Germany). Washed suspended cells were incubated with EpCAM-APC (Miltenyi Biotech, Cat. No. 120-001-554) and CD90-biotin- (Beckman Coulter, Miami, FL, USA, Cat. No. 559944) labeled antibodies for 15 min at 4 °C, rigorously washed three times in PBS-A buffer with 2 mmol/l EDTA. CD90+ cells were labeled with Streptavidin-ECD (Beckman Coulter, Cat. No. PN IM3326), and resuspended to a concentration of 100 million cells in 300 μl of PBS—2 mmol/l EDTA containing 0.5% bovine serum albumin (Blue Buffer). EpCAM/CD90 stained cell suspensions were labeled according to the manufacturer’s instructions with anti-APC (Miltenyi Biotech, Cat. No. 130-090-855) and anti-PE microbeads (Miltenyi Biotech, Cat. No. 130-048-801) and gently mixed in a MACSmix tube rotator (Miltenyi Biotech) for 15 min in the refrigerator (6 °C). Preliminary experiments determined that anti-PE microbeads bind to ECD-labeled cells. ECD is a trade name for PE-Texas Red. Microbead-labeled cells were washed carefully two times with 50 ml of ice-cold sterile Blue Buffer and resuspended to a concentration of 100 million per 0.5 ml of Blue Buffer. Labeled cells were divided into 0.5-ml aliquots of 100 million cells each which were loaded serially into the dual column separation set up, followed by a 2-ml sample tube wash of Blue Buffer. An average of 2×10^9^ viable cells was loaded per sample. EpCAM+ and CD90+ cells were enriched using the POSSELDS selection program, which is designed to maximize the recovery of labeled cells at the expense of purity. Both, EpCAM- and CD90-positive and -negative fractions were collected. The purity of isolated cells was determined by flow cytometric analysis detecting the APC-labeled EpCAM and ECD-labeled CD90 antibodies.

### Staining and flow cytometry

In order to minimize nonspecific binding of fluorochrome-conjugated antibodies, pelleted cell suspensions were preincubated for 5 min with neat decomplemented (56 °C, 30 min) mouse serum (5 μl).^26^ Prior to intracellular cytokeratin staining, cells were stained for surface markers (2 μl each added to the cell pellet, 15–30 min on ice); CD44-PE (AbD Serotec, Cat. No. MCA89PE), CD90-biotin (BD, Cat. No. 555594), Streptavidin-ECD (Beckman Coulter, Cat. No. IM3326), CD117-PE-Cy7 (BD, Cat. No. IM3698), lineage-PE-Cy5 cocktail ([CD14, CD33, Glycophorin A]-PE-Cy5, BD, Cat. No. IM2614, IM26470, 559944), EpCAM-APC (Miltenyi Biotech, Cat. No. 130-091-254), CD45-APC-Cy7 (BD, Cat. No. 37629), CD146-APC-Alexa700 (BD, Cat. No. 6699531), and fixed with 2% methanol-free formaldehyde (Polysciences, Warrington, PA, USA). Cells were then permeabilized with 0.1% saponin (Beckman Coulter) in phosphate-buffered saline with 0.5% human serum albumin (10 min at room temperature). Permeabilized cells were pelleted by centrifugation at 400*g* for 7 min at room temperature, supernatant was discarded and cell pellets were incubated with 5 μl of neat mouse serum for 5 min, centrifuged, and decanted. The cell pellet was disaggregated and incubated with 2 μl of anti-pan cytokeratin-FITC (Beckman Coulter, Cat. No. IM2356) for 30 min. Cells pellets were diluted to a cell concentration of 10 million cells per 400 μl of staining buffer and DAPI (Sigma-Aldrich, Cat. D1306) was added to a final concentration of 7.7 μg/ml.

Nine-color analysis was performed using the 3-laser, 10-color Gallios cytometer (Beckman Coulter). Samples were acquired exhaustively (range=0.8–4.9×10^5^ events) at rates not exceeding 10,000 events per s. DAPI was acquired in two fluorescence channels (FL9 and FL10), with PMT gain optimized for linear (cell cycle), and log (elimination of hypodiploid events) acquisition, respectively. The cytometer was calibrated to predetermined photomultiplier target channels prior to each use using eight-peak Rainbow Calibration Particles (Spherotech, Libertyville, IL, USA, Cat. No. RCP-30-5A). Offline compensation and analysis were performed using VenturiOne, an analytical package utilizing scalable parallel processing and designed specifically for multiparameter rare event problems (Applied Cytometry, Dinnington, Sheffield, UK). Spectral compensation matrices were calculated for each staining combination within each experiment using single-stained mouse IgG capture beads (BD Biosciences, San Jose, CA, USA, Cat. No. 552843) for each tandem dye and BD Calibrite beads for FITC, PE and APC controls.

### Cell lines

BT474 cells were purchased from ATCC and passaged according to instruction. ASC were isolated from the stromal vascular fraction of human adipose tissue and expanded in culture as previously described.^27,28^ Cells were used in this study at passage 1.

### Xenograft injections

The experimental protocols were approved by the University of Pittsburgh’s Institutional Animal Care and Use Committee (IACUC, protocol number 0909770) and were performed in strict accordance with the recommendations in the Guide for the Care and Use of Laboratory Animals of the National Institute of Health.^29^ Twenty-seven female NOD-*scid Il2rg*^*null*^ mice (NOD.Cg-Prkdc^Scid^Il2rg^tmlWjl/SzJ^, Jackson Lab, Bar Harbor, ME, USA, Cat. No. 005557) 6–8 weeks of age, weighing 19.48±1.31 g, were purchased from The Jackson Laboratory, and housed five to a cage in a specific pathogen-free environment. Before injection of tumor cells, mice were anesthetized by methoxyflurane inhalation. Animals receiving immunomagnetically sorted cells (*n*=15) received a total of four injections in the mammary fat pads (MFP). Each 50-μl injection consisted of 25 μl immunomagnetically enriched EpCAM+ and/or CD90+ cells (0.273–1.649×10^6^ cells per injection, 1/20 of total recovered cells) in combination with 1.0×10^5^ human adipose stromal cells (ASC, 080607, passage 1) in ice-cold Dulbecco's Modified Eagle Medium, 15% FBS and 25 μl Matrigel (356234; BD Biosciences) per injection. Control animals (*n*=12) received ASC in Matrigel, whereas positive control animals were similarly injected with graded numbers of BT-474 cells (10 and 1 million cells per injection) in 50 μl Matrigel. Animals were examined twice weekly for behavioral changes and evidence of tumor growth. Mice were killed 6 weeks (BT-474 group) or 6 months postinjection. Collected tissues were fixed in 10% neutral buffered formalin (Sigma-Aldrich, Cat. No. F5554). Paraffin embedding and sections (4–5 μm) were prepared at the McGowan Institute histology laboratory.

### Histologic evaluation

Tissue microsections were deparaffinized in xylenes and rehydrated with a graded series of ethanol. The tissue sections were stained with hematoxylin and eosin (H&E), dehydrated and mounted in xylene mounting medium.

### Immunofluorescent staining of paraffin-embedded tissues

Tissue microsections were deparaffinized in xylenes and rehydrated with a graded series of ethanol. Heat-mediated antigen retrieval was performed using Dako Target Retrieval Solution at pH6 (20 min, 125 °C) in a Pascal pressure chamber (Dako, Carpinteria, CA, USA). Tissue sections were washed twice in Dako Wash Buffer and then incubated for 1 h in blocking solution (PBS, 5% goat serum, 0.05% Tween 20) to reduce nonspecific antibody binding. Blocking solution was used for all subsequent antibody dilution. Primary antibodies were directly applied to tissue sections. Primary antibodies were incubated overnight at 4 °C. Primary antibodies were mouse anti-human Ki-67 (clone MIB-1, #M724029-2, DAKO, 1:100), rabbit anti-Histone H1 (clone EPR6537, #ab125027, Abcam, 1:100), and rat anti-mouse major histocompatibility complex class 1 (clone ERMP42, #ab15680, Abcam, Cambridge, UK, 1:100). Universal Negative Control for Mouse Primary Antibodies (ready to use, N1698, Dako) and Universal Negative Control for Rabbit Primary Antibodies (ready to use, N1699 Dako) substituted primary antibodies for negative controls. Slides were washed three times in wash buffer and incubated for 1 h with a secondary biotinylated goat anti-mouse and biotinylated goat anti-rabbit antibody solution (both 1:500, Dako) or biotinylated goat anti-rat antibody solution (Vector Laboratories, Burlingame, CA, USA, 1:500). Slides were washed twice and incubated for 30 min with streptavidin-Cy3 (1:500, Sigma). Samples were washed three times and, where applicable, counterstained for 1 h at ambient temperature with FITC-conjugated mouse anti-human pan cytokeratin antibody (clone AE1/AE3, 53-9003-80, eBioscience, 1:100). Sections were washed twice and incubated for 5 min at ambient temperature with 7.15 μM DAPI solution for nuclear staining. Slides were washed twice in phosphate-buffered saline and mounted using Prolong Gold Antifade Reagent (P36934, Invitrogen, Carlsbad, CA, USA). Photomicrographs were taken using a Nikon Eclipse 800 Microscope (Melville, NY, USA) equipped with a CCD camera.

## Figures and Tables

**Table 1:** Mobilization regimens

*URN*	*Chemo*	*Dose per day*	*Days*	*G-CSF mobilization*	
				*Dose (μg/kg per day)*	*Days*
10-014	Cyclophosphamide	5 g/m^2^	1	5	16
10-015	None			15	9
	Cyclophosphamide	1,500 mg/m^2^			
10-016	Thiotepa	125 mg/m^2^	4	5	21
	Carboplatin	200 mg/m^2^			

Abbreviation: G-CSF, granulocyte colony stimulating factor. Hematopoietic progenitor cells were mobilized into the peripheral blood with daily doses of G-CSF beginning 1 day after the last dose of chemotherapy. Products were collected when the white blood corpuscles were ⩾1,000 cells per μl for 3 consecutive days.

**Table 2:** Distribution of cells according to their EpCAM or CD90 surface marker expression following immunomagnetic cell enrichment

*URN*	*Cell population*	*Nucleated cells collected (%)*	*Cell purity of EpCAM+ and/or CD90+ cells (%)*
10-014	EpCAM+/CD90+	5.5	
	EpCAM+/CD90−	93.0	
	EpCAM−/CD90+	0.5	
	EpCAM−/CD90−	1.0	98.9
10-015	EpCAM+/CD90+	5.1	
	EpCAM+/CD90−	38.9	
	EpCAM−/CD90+	35.3	
	EpCAM−/CD90−	20.7	79.1
10-016	EpCAM+/CD90+	2.7	
	EpCAM+/CD90−	90.0	
	EpCAM−/CD90+	1.3	
	EpCAM−/CD90−	6.0	93.9

**Table 3:** Frequency of palpable tumors after injection of enriched circulating EpCAM+ tumor cells (CTCs) or BT-474 in to the mammary fat pad of NOD-*scid Il2rg^null^* mice

*ID*	*No. of cells injected per site*	*No. of ASC injected per site*	*Frequency of tumors per site*
URN10-014^a^	0.3×10^6^	10,000	2/20^b^
URN10-015^a^	1.3×10^6^	10,000	0/16^c^
URN10-016^a^	1.2×10^6^	10,000	0/20
BT-474^d^	1,000,000	10,000	5/5
	100,000	10,000	5/5
	10,000	10,000	4/5
	1,000	10,000	3/5
	100	10,000	2/5
	10	10,000	1/5
Feeder cells only^a^	—	10,000	0/18
PE16 CD90+ (high light scatter)	100	10,000	9/20
PE30 CD90+ (high light scatter)^e^	100	10,000	8/20

Abbreviation: ASC, adipose stromal cells.

a 6 Months after injection.

b Both tumors were of lymphoid morphology and confirmed to be of murine origin.

c One animal was not evaluable due to early death unrelated to tumor injection.

d 6 Weeks after injection.

e Previously published data (Zimmerlin *et al.*, Tissue Engineering Part A, 2011); PE16 and PE30 were unpassaged FACSorted breast cancer metastatic pleural effusions.

Animals were killed at 6 months and 6 weeks, respectively.
